# Pea-Tea Intercropping Improves Tea Quality through Regulating Amino Acid Metabolism and Flavonoid Biosynthesis

**DOI:** 10.3390/foods11223746

**Published:** 2022-11-21

**Authors:** Qingping Ma, Laichao Song, Zhanhai Niu, Ziyuan Qiu, Haiwei Sun, Zhihong Ren, Huanhuan Wu, Yu Wang, Huiling Mei, Xinghui Li, Zhaotang Ding

**Affiliations:** 1 College of Agronomy, Liaocheng University, Liaocheng 252000, China; 2 Taian Academy of Agricultural Sciences, Taian 271000, China; 3 College of Horticulture, Qingdao Agricultural University, Qingdao 266109, China; 4 International Institute of Tea Industry Innovation for “one Belt, one Road”, Nanjing Agricultural University, Nanjing 210095, China; 5 Tea Research Institute, Shandong Academy of Agricultural Sciences, Rizhao 276800, China

**Keywords:** intercropping, amino acid biosynthesis, catechins, flavonoids, tea quality

## Abstract

Pea-tea intercropping is an excellent cultivation method that can improve tea quality. However, the underlying mechanism is still unclear. The present study was aimed at elucidating the mechanism of the effect of pea-tea intercropping on tea quality through a high-throughput method. Transcriptome and metabolome analyses were conducted to identify the changes in gene expression and metabolites changes intercropping, respectively. In addition, the amino acids and catechins were detected using the LC-MS method and quantified absolutely. The results showed that total polyphenols and catechins decreased but amino acids increased in pea intercropped tea shoots. Correspondingly, genes related to amino acid metabolism and flavonoid biosynthesis were differentially expressed. For amino acid metabolism, 11 differentially expressed genes were identified, including 5 upregulated and 6 downregulated genes. Meanwhile, three genes involved in carbohydrate transport and metabolism were upregulated in pea intercropped tea plants. These genes were also involved in amino acid metabolism. For flavonoid biosynthesis, two downregulated genes were identified, which were the flavonol synthase and anthocyanidin synthase genes and followed a similar pattern to changes in catechins and polyphenols. These advances have opened new horizons for understanding the biochemical mechanisms of amino acids and flavonoids in improving tea quality in the pea-tea intercropping cultivation model.

## 1. Introduction

Tea plant (*Camellia sinensis*) is an evergreen shrub or tree that is distributed in subtropical and tropical regions. New shoots of the tea plant is the source of the popular drink, tea. The growth and quality of tea shoots are affected by many factors, such as soil nutrition, environmental factors, and cultivation methods.

In order to promote the growth and improve the quality of tea, intercropping has been widely used in recent years. Many intercropping plants have been reported to be beneficial for promoting the growth of tea plants and improving tea quality, which includes woody and herbaceous plants. For woody plants, Chinese chestnut-tea intercropping could improve the tea’s quality by promoting amino acid biosynthesis, which contributes to the umami taste of tea, and reduce the bitter taste associated with flavonoids. In addition, some other protective factors, such as allantoic acid, sugars, and unsaturated fatty acids, were higher in Chinese chestnut intercropped tea than mono-cropped tea [1]. Meanwhile, the soil nutrients, including nitrogen, phosphorus, potassium, and organic matter, were increased in the Chinese chestnut-tea intercropping field, and some beneficial bacteria were enriched in the intercropped soil [2]. In the rubber intercropped tea garden, the land expectation value was significantly higher than that in rubber or tea mono-cropped gardens, and the prices for rubber and tea were also improved [3]. Except for soil nutrients, walnut-tea intercropping could increase bacterial and fungal diversity [4]. In addition, some other woody plants, such as persimmons, were usually used for intercropping with tea plants [5]. These plants can not only protect tea plants against the strong light stress in the summer but also improve soil nutrient availability, soil enzyme activity, and tea quantity and quality [6]. 

For herbaceous plants, aromatic plants, such as *Cassia tora* and *Leonurus artemisia* could obviously reduce the population levels of leafhoppers (*Empoasca onukii* Matsuda) [7]. Maize (*Zea mays*) could provide shade for tea plants, improve tea quality, and change soil microorganism diversity [8]. Nevertheless, leguminous plants are the most popular intercropping plants used in tea gardens. For example, soybean-tea intercropping could regulate the amino acid metabolism and secondary metabolism of tea leaves and improve the total nitrogen in the soil of a tea garden [9,10,11,12]. Peanut (*Arachis hypogaea*) could improve soil organic carbon and soil nutrient availability and affect soil enzymatic responses at different depths, especially increasing the activity of acid phosphatase, protease, and sucrose [13]. However, these leguminous plants are annuals with only a 5-month life cycle from June to October. Therefore, other cold-tolerant leguminous plants are required. Pea (*Pisum sativum*) is also an annual leguminous plant that is planted in winter and harvested in summer. It is optimal for rotation with soybeans. In addition, as with other leguminous plants, peas could also fix nitrogen, which is an important macroelement for plant growth. Thus, pea intercropping could provide nutrients for tea plants in the spring of the tea harvest season. However, the molecular mechanism of pea intercropping on tea plants remains unclear.

In order to illustrate the effect of pea-tea intercropping on the growth and quality of tea shoots, the transcriptome of tea shoots from monocropped and pea intercropped tea plants was analyzed using Illumina high throughput sequencing to screen the key genes involved in various metabolic pathways regulated by intercropping. The metabolome was also generated by ultra high-performance liquid chromatography-tandem mass spectrometry (UPLC-MS) for evaluating the effect of intercropping on tea quality. In addition, target analysis of some chemicals, including amino acids, polyphenols, and catechins in tea shoots, was performed to evaluate the role of pea-tea intercropping on the metabolism of flavonoids and amino acids. This study will guide the selection of intercrop plants for tea plants and also provide scientific significance for understanding the interaction mechanisms of tea plants and their intercropping plants. 

## 2. Materials and Methods

### 2.1. Plant Materials 

This study was conducted in the Jiushengyudao tea garden, which is located in Changqing District, Shandong Province, China (36°16′ N, 116°59′ E, 200 m above sea level). Three year old tea plants (*Camellia sinensis* cv. *Zhongcha108*) were intercropped with peas. After flowering of pea, tea shoots with one bud and two tender leaves were harvested and stored in −80 °C for RNA and metabolite extraction. Monocropped tea plants were used as controls. Three replicates were performed for each sample.

### 2.2. RNA Extraction and cDNA Library Construction

Total RNA was extracted using the Plant Quick RNA isolation Kit (Huayueyang Biotech Ltd., Beijing, China) following the manufacturer’s instructions. RNA integrity and concentration was evaluated using a NanoDrop 2000 (Thermo Fisher Scientific, Waltham, DC, USA), Agilent Bioanalyzer 2100 system (Palo Alto, CA, USA), and 1% agarose gel electrophoresis. A total of 1 μg RNA was used for cDNA library construction using the NEBNext UltraTM RNA Library Prep Kit (Ipswich, MA, USA). Briefly, magnetic beads containing Oligo(dT) were used to enrich the mRNA. A fragmentation buffer was added for interrupting the mRNA into fragments. The first strand of cDNA was synthesized by reverse transcription using random hexamers, and the second strand of cDNA was synthesized by adding reaction buffer, dNTPs, RNase H, and DNA polymerase I. The final product was purified by AMPure XP beads. After repairing the ends, adding poly-A, and connecting the sequencing adapter, the size of the fragments was identified by AMPure XP beads. A cDNA library was obtained after PCR analysis. The quality of the cDNA library was detected by the Agilent Bioanalyzer 2100 system.

### 2.3. Transcriptome Sequencing and Data Assessment

The high-quality cDNA library was clustered using the TruSeq PE Cluster Kit v4-cBot-HS (Illumina) and sequenced on the Illumina 4000 platform by BioMarker Biotech Ltd. (Beijing, China). Paired-end raw data was generated. The clean data was obtained after removing low-quality reads containing adapters, reads with ploy-N over 10%, and reads with a Q **≤** 10 over 50% from the raw data. The clean reads with Q30 over 85% were used for further analysis. 

### 2.4. Differentially Expressed Genes Identification and Function Annotation

The clean reads were mapped to the tea genome (http://tpdb.shengxin.ren/, accessed on 6 May 2020) using HISAT 2.0.4 [14]. The assembly and expression evaluation of mapped reads were analyzed using the StringTie v.1.3.4 software (Baltimore, MD, USA) [15]. Gene expression was evaluated based on fragments per kilobase of transcript per million fragments mapped (FPKM) [16]. Fold change of *FPKM* ≥ 2 and a false discovery rate (FDR) < 0.05 were used as the threshold values for screening differentially expressed genes (DEGs). The DEGs were annotated to Gene ontology, the Cluster of Orthologous Groups of roteins (COG), the Kyoto Encyclopedia of Genes and Genomes (KEGG), Swiss-Prot, and NR databases by using the DIAMOND v.2.0.4 software (Bonn, Germany) [17]. The biological variability of the expression of DEGs between samples was assessed using Pearson’s correlation coefficient. The closer R^2^ is to 1, the stronger the correlation between samples. 

### 2.5. Quantitative Real-Time PCR Verification

To verify the reliability of expression of DEGs, 8 DEGs were selected randomly for quantitative real-time PCR (qRT-PCR) analysis. The qRT-PCR was performed using a BioRad CFX96 real time detection system in a 20 μL reaction volume consisting of 10 μL of the SYBR Green qPCR Mix (Biosharp, Hefei, China), 1 μL of primers (10 μM), and 2 μL of cDNA with the following reaction conditions of 95 °C for 5 min, followed by 40 cycles of 95 °C for 10 s and 60 °C for 30 s. A RT reaction mix without reverse transcriptase served as the negative control. *CsGAPDH* was used as the reference gene (Table S1). The relative expression of the selected genes was calculated using 2^−ΔΔCT^ method [18]. 

### 2.6. Metabolite Extraction and Metabolome Analysis

Metabolite extraction and analysis were conducted as in the previous study by Ma et al. [19]. The samples were freeze-dried in the Scientz-100F lyophilizer (Shenzhen, China). A total of 100 mg of powder was resolved in 1.2 mL of 70% methanol overnight at 4 °C. After centrifugation for 10 min at 12,000 rpm, the supernatant was filtered using a 0.22 μm membrane and used for UPLC-MS analysis. 

Four microliter liquid extracts were injected into a Shimadzu UPLC system containing an Agilent SB-C18 column (1.8 µm, 2.1 mm × 100 mm). Pure water and acetonitrile with 0.1% formic acid were used as mobile phases A and B, respectively. The following gradient was used: 5% B at 0 min, increased to 95% in 9 min and kept for 1 min, decreased to 5% in 1 min and kept for 3 min. The total flow of mobile phases was 0.35 mL/min. The column temperature was kept at 40 °C. 

The parameters for the electrospray ionization source were: 550 °C, 5500 V (+)/4500 V (−), 50 psi (GSI), 60 psi (GSII), and 25 psi (CUR). The collision-induced ionization parameter was set to high. The ions were scanned by triple quadrupole mass spectrometry (QQQ) using the multiple reaction monitoring method (MRM). The qualitative analysis of metabolites was performed using secondary mass spectrometry information. Quantitative analysis of metabolites was performed using MRM and the peak area normalization methods. Fold change > 1, VIP ≥ 1, and a *p* < 0.05 were used as the thresholds for selection of differential metabolites. The Spearman Rank Correlation analysis was used to assess the biodiversity of samples, with an R^2^ close to 1 indicating a high correlation. Functional annotation of the metabolites was conducted using the KEGG database.

### 2.7. Detection of Tea Quality Components 

Tea polyphenols were detected by a tea polyphenols detection kit (Yuanye Biotech Ltd., Shanghai, China) using the folinol colorimetric method according to the manufacturer’s introductions. Catechins were detected using UPLC analysis according to the Chinese national standard GB/T 8313-2018. Briefly, 0.2 g of tea powder was extracted by a 10 mL 70% methanol solution at 70 °C for 10 min. The solution was centrifuged at 5000 rpm for 10 min, and the liquid supernatant was filtered using a 0.22 µm membrane. A total of 10 µL of supernatant was injected in the UPLC system with the SPD-M30A detector (SHIMADZU, Japan) and C18 column (Waters, 5 µm, 250 mm × 4.6 mm). A pure water and acetonitrile solution containing 5% acetic acid and 0.5% EDTA-Na were used as mobile phases A and B, respectively. An isocratic elution procedure was performed with a total flow of 0.5 mL/min. The column temperature was maintained at 35 °C. Pure catechins (>98%), including gallic acid (GA), catechin (C), epicatechin (EC), gallocatechin (GC), epigallocatechin (EGC), gallocatechin gallate (GCG), epigallocatechin gallate (EGCG), catechin gallate (CG), and epicatechin gallate (ECG) were purchased from Yuanye Biotech Ltd. (Shanghai, China) and used as the standard for qualitative analysis.

A total of 94 target amino acids and derivates were detected by Metware Biotech Ltd. (Wuhan, China) using the UPLC-MS method. Briefly, 50 mg of tea powder was added in 500 µL of cold 70% methanol and centrifuged at 12,000 rpm for 10 min at 4 °C, and centrifuged again after cooling for 30 min at −20 °C. Two microliter liquid supernatant was injected in the Liquid Chromatography (ExionLC™ AD) with an ACQUITY BEH Amide column (1.7 µm, 100 mm × 2.1 mm). Pure water and acetonitrile containing 2 mM ammonium acetate and 0.4% methanoic acid were used as mobile phases A and B, respectively. Threonine-D2 (1 μg/mL) was used as the internal standard. The following gradient procedure was performed: 10% A in 1.2 min, increased to 40% A at 9 min, increased to 60% A at 10 min and kept for 1 min, then decreased to 10% and kept for 4 min. The flow rate was 0.4 mL/min, and the column temperature was kept at 40 °C. Tandem mass spectrometry (QTRAP^®^ 6500+) was used for the identification of amino acids under the following conditions: electrospray ionization at 550 °C, 5500 V in positive ion mode, and 35 psi curtain gas. Qualitative analysis of amino acids was conducted using a pure standard and the MWDB database (Metware). The content of amino acids was calculated based on the standard curves. In order to display the difference in amino acids content between mono- and inter-cropped tea, Z-score normalization was conducted according to the following formula:

χ’ = (X − μ)/σ(1)

(X: content of amino acids, μ: mean value, σ: standard deviation)

### 2.8. Statistical Analysis

Statistical analysis was performed by using Microsoft Excel 2016 and SPSS 23.0. The difference between groups was analyzed by using a *t-*test and a *p* < 0.05 was considered to be statistically significant. Cluster analysis of amino acids was performed by using the ComplexHeatmap package v.2.7.1.1009 in the R software v.3.5.1(Seattle, WA, USA) [20].

## 3. Results

### 3.1. Tea Quality Components in Pea-Tea Intercropping Plants

**Figure 1:**
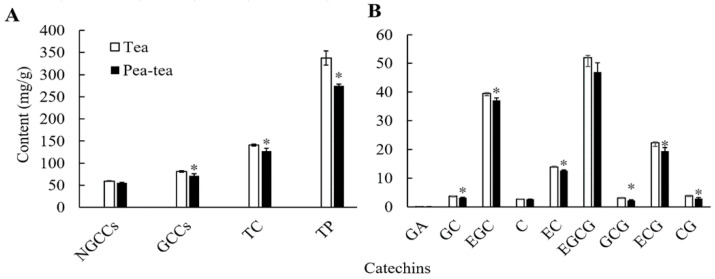
Tea catechins in new shoots from pea-tea intercropping plants. Asterisks * represents significant differences compared to monocropped tea shoots. NGCCs: non-gallocatechins, GCCs: gallocatechins, TC: total catechins, TP: tea polyphenol, GA: gallic acid, GC: gallocatechin, EGC: epigallocatechin, C: catechin, EC: epicatechin, EGCG: epigallocatechin gallate, GCG: gallocatechin gallate, ECG: epicatechin gallate, and CG: catechin gallate.

Tea polyphenols and catechins were important tea quality components and played important roles in the taste of tea. Tea polyphenol comprises 337.34 mg/g and 275.00 mg/g in new shoots from monocropped and pea intercropped tea plants, respectively. Compared to monocropping of tea plants, the total polyphenol content was significiantly decreased (*p* < 0.05) in new shoots from pea intercropped tea plants (Figure 1A). Overall, the total catechins and gallocatechins (GCCs) were significantly lower in intercropped tea shoots compared to monocropped tea. For monomer catechins, EGCG accounted for the highest levels in tea (52.03 mg/g and 47.01 mg/g in mono and intercropped tea, respectively)*,* followed by EGC (39.53 mg/g and 37.10 mg/g in mono and intercropped tea, respectively), and ECG (22.27 mg/g and 19.36 mg/g in mono and intercropped tea, respectively). Of these catechins, three gallocatechins (GCG, ECG, and CG) and two non-gallocatechins (GC and EC) were higher in monocropping tea plants than in intercropping tea plants (Figure 1B). The results indicated that pea-tea intercropping could decrease the polyphenol and catechin contents of tea plants.

### 3.2. Quality of Transcriptomic Data

A total of 47.72 GB of clean data was obtained from six tea samples. All the samples showed a Q30 of 94.72–95.49%, indicating high-quality transcriptomic data. More than 87.73% clean reads were mapped to the tea genome, and over 74.04% unique mapped reads were produced (Table 1). The clean reads were uploaded to the SRA database with the accession number PRJNA861687.

### 3.3. Identification of Differentially Expressed Genes 

**Figure 2:**
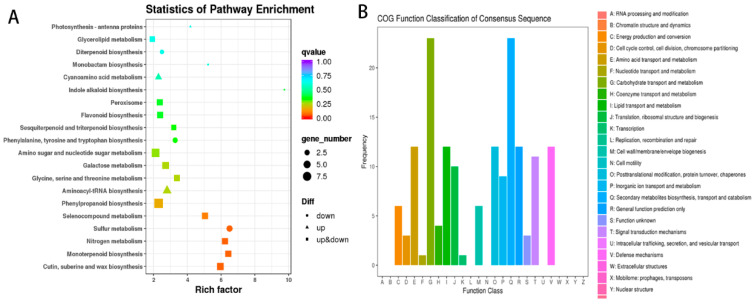
Functional annotation of differentially expressed genes (DEGs) between monocropped and pea intercropped tea plants. (**A**) KEGG pathway enrichment analysis of DEGs; q-value indicate degree of enrichment of DEGs, the lower of q-value, the greater the degree of enrichment; (**B**) COG function classification of DEGs.

Correlation analysis revealed that biological replicates in both mono- and inter-cropping samples had a strong correlation (R^2^ > 0.99). Thus, the differential expression analysis was reliable. A total of 304 DEGs, including 138 upregulated DEGs and 166 down-regulated DEGs, were identified in intercropped tea plants. Of these, 299 DEGs were annotated in the NR database. In addition, 280, 259, 252, 235, 206, 140, and 129 DEGs were identified in the eggNOG, Pfam, GO, Swiss-Prot, KEGG, KOG, and COG databases, respectively. KEGG pathway enrichment analysis showed that sulfur metabolism, nitrogen metabolism, monoterpenoid biosynthesis, cutin, suberine, and wax biosynthesis possessed the highest enrichment (Figure 2A). COG function classification analysis showed that “Carbohydrate transport and metabolism” and “Secondary metabolites biosynthesis, transport and catabolism” enriched the most DEGs (Figure 2B). 

**Figure 3:**
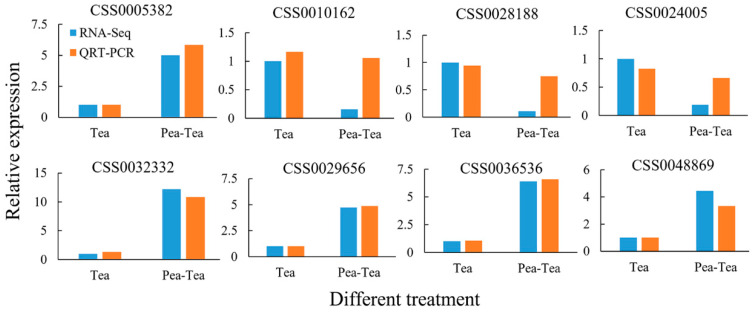
Quantitative real-time PCR verification of differentially expressed genes. RNA-Seq indicates fold change of *FPKM* and QRT-PCR means relative expression of *FPKM* cDNA as calculated by 2^−ΔΔCT^ method.

qRT-PCR verification revealed that the relative expression of all selected DEGs showed similar expression trends with fold changes of *FPKM* from RNA-Seq analysis (Figure 3). The result indicated that DEG identification based on RNA-Seq analysis was reliable and could be used in further analyses.

### 3.4. Differential Metabolite Identification

**Figure 4:**
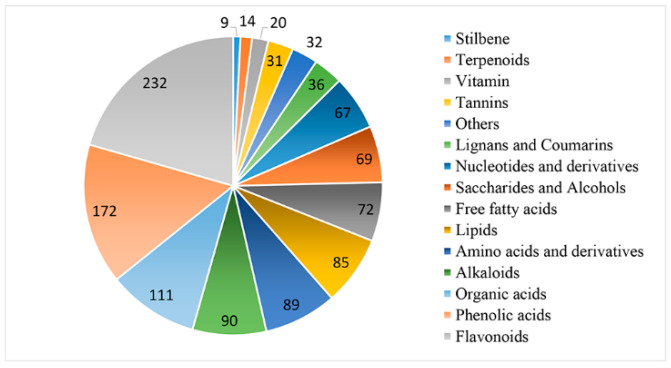
The composition of and metabolites identified in tea shoots.

A total of 1129 metabolites were detected in tea plants. Flavonoids were the richest metabolites in tea plants, including 232 compounds. Phenolic acids are the second class, accounting for 172 compounds. In addition, organic acids (111), alkaloids (90), amino acids and derivates (89), lipids (85) and free fatty acids (72), saccharides and alcohols (69), nucleotides and derivates (67), lignans and coumarins (36), tannins (31), vitamins (20), terpenoids (14) and stilbene (9) were the major metabolites in tea shoots (Figure 4).

**Figure 5:**
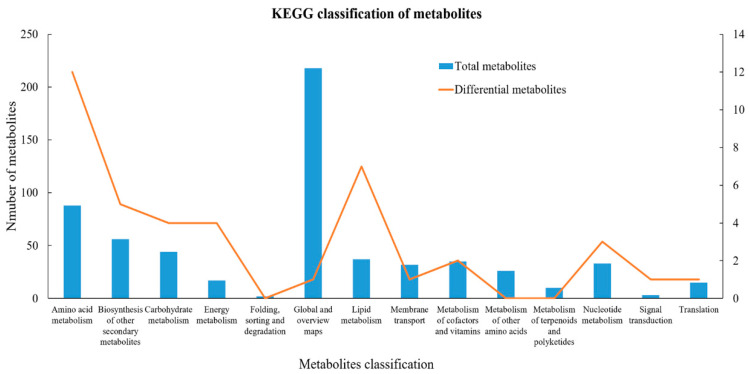
KEGG annotation analysis of metabolites in pea intercropped tea plants.

A total of 616 metabolites were annotated in the KEGG database. Global and overview maps (288), amino acid metabolism (88), and biosynthesis of secondary metabolites (56) enriched the most metabolites. Compared to monocropped tea plants, pea intercropped tea plants had 196 differential metabolites, including 114 up-regulated and 82 down-regulated metabolites. Of these, 41 differential metabolites were annotated in the KEGG database. Amino acid metabolism (12), lipid metabolism (7), and biosynthesis of secondary metabolites (5) enriched the most differential metabolites (Figure 5 and Table S2). Of these differential metabolites, most amino acids and lipids, especially free fatty acids were higher in pea intercropped tea than that in monocropped tea. Amino acids contributed to the umami taste of tea, and lipids were the precursors of volatile matters, which formed the pleasant tea aroma. Flavonoids are the major secondary metabolites in tea shoots, which showed antioxidant effects, and some of them exhibited a bitter taste. In this study, the total polyphenols and catechins were decreased in pea intercropped tea shoots. Therefore, pea-tea intercropping improved tea quality by promoting amino acid and lipid biosynthesis while suppressing flavonoid biosynthesis to some extent. 

### 3.5. Amino Acid Metabolism in Pea Intercropped Tea Plants

**Figure 6:**
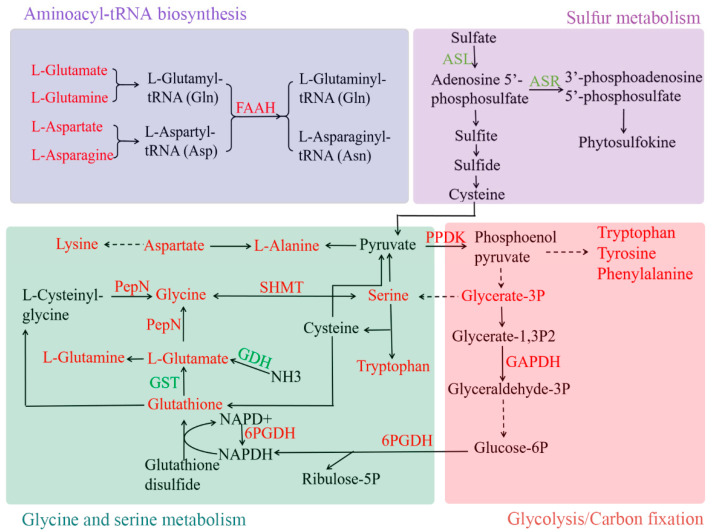
The amino acid metabolism in pea intercropped tea shoots. The red and green colored text indicate up- and down-regulated genes or metabolites, respectively.

In this study, 11 amino acid metabolism related to DEGs were identified. Of these, serine hydroxymethyltransferase (SHMT, CSS0006418, 0.60), puromycin-sensitive aminopeptidase (PepN, CSS0019820, 0.75), and prolyl endopeptidase (PEP, CSS0037504, 1.16) were up-regulated in pea intercropped tea plants, whereas three chloroplastic 5’-adenylylsulfate reductase 1 genes (ASRs, CSS0028007 −0.83, CSS0030143 −1.07 and CSS0009987 −0.83), the ATP sulfurylase gene (ASL, CSS0042606 −1.01), glutathione S-transferase gene (GST, CSS0010169 −0.99), and the glutamate dehydrogenase gene (GDH, CSS0034454 −1.28) were down-regulated. In addition, two fatty acid amide hydrolase genes were up-regulated in pea intercropped tea plants (FAAH, CSS0001960 0.72, and CSS0017124 0.91), which were aminoacyl-tRNA biosynthesis related genes (Figure 6).

**Figure 7:**
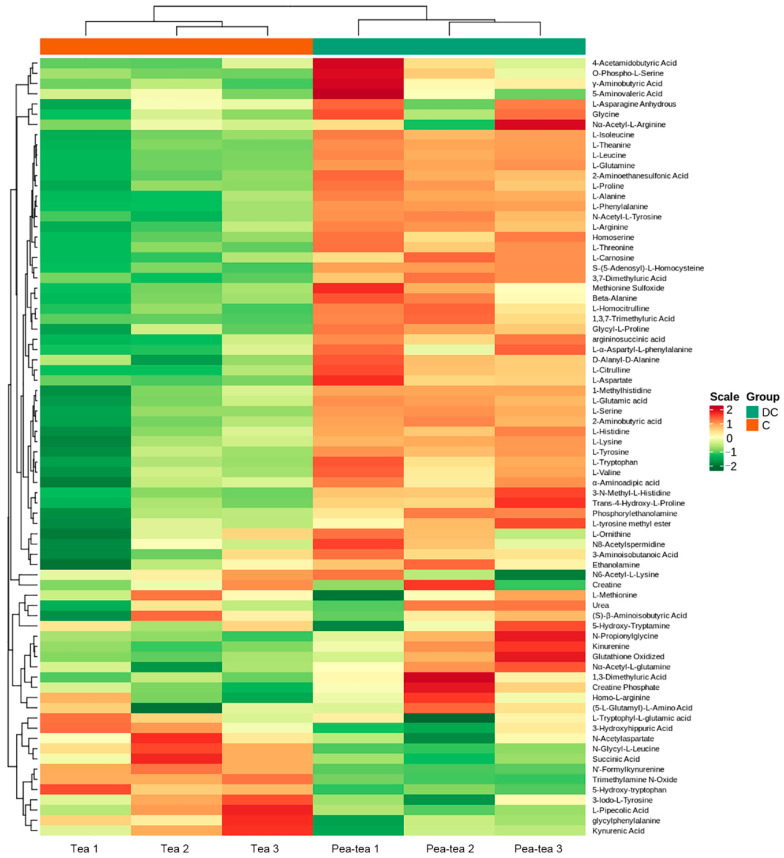
Heatmap of amino acid changes in monocropped and pea intercropped tea plants.

There were 76 amino acids and derivates identified in tea shoots. These amino acids account for 45.23 mg/g in monocropped tea shoots and 50.57 mg/g in pea intercropped tea shoots (Table S3). This result indicated that pea-tea intercropping could improve the total amino acids of tea shoots. Most of the amino acids showed higher content in pea intercropped tea plants than in monocropped tea, such as oxidized glutathione (11.15 mg/g in monocropped tea and 14.25 mg/g in pea intercropped tea), L-glutamate (Glu, 5.41 mg/g in monocropped tea and 5.93 mg/g in pea intercropped tea), L-glutamine (Gln, 1.38 mg/g in monocropped tea and 1.75 mg/g in pea intercropped tea), and L-theanine (0.57 mg/g in monocropped tea and 0.62 mg/g in pea intercropped tea), which were the major amino acids contributing to the umami taste of tea (Figure 7 and Table S3). 

### 3.6. Carbohydrate Transport and Metabolism in Pea Intercropped Tea Plants

Three carbohydrate transport and metabolism-related genes, including glyceraldehyde 3-phosphate dehydrogenase (GAPDH, CSS0022957 0.66), 6-phosphogluconate dehydrogenase (6PGDH, CSS0000260 4.81), and pyruvate phosphate dikinase (PPDK, CSS0033563 1.10), showed higher expression levels in pea intercropped tea plants than in monocropped tea plants. Meanwhile, glycerate-3-phosphate (1.54) and sedoheptulose (1.51) were accumulated in pea intercropped tea plants (Figure 6). 

### 3.7. Flavonoid Metabolism in Pea Intercropped Tea Plants

**Figure 8:**
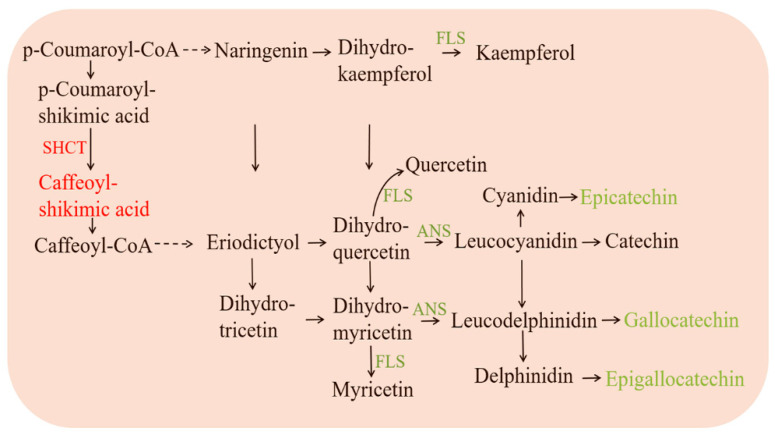
Flavonoid metabolism in pea intercropped tea plants. The red and green colored text indicate up- and down-regulated genes or metabolites, respectively.

An anthocyanidin synthase gene (ANS, CSS0011888, −5.49) and a flavonol synthase gene (FLS, CSS0007481, −4.02) were down-regulated in the pea intercropped tea plant, which were related to the biosynthesis of catechins. The expression of both genes was consistent with the change in catechin and total polyphenol content. In pea intercropped tea plants, the shikimate O-hydroxycinnamoyl transferase gene (SHCT, CSS0002667 0.59) was upregulated (Figure 8). 

In pea intercropped tea plants, a total of 31 differential flavonoids were identified, including 23 decreased flavonoids and 8 increased flavonoids (Table S2). To some extent, flavonoid biosynthesis was suppressed because of the reduced flavonoids. For these differential flavonoids, only catechins and caffeoyl shikimic acids were annotated in the KEGG pathway. Except for caffeoyl shikimic acid, most catechin and total polyphenol content were decreased in pea intercropped tea shoots. 

## 4. Discussion

To elucidate the effect of pea intercropping on the metabolism of tea plants, the transcriptome, metabolome, and tea quality-associated metabolites for monocropped and pea intercropped tea plants were detected. The results indicated that amino acid metabolism was promoted in pea intercropped tea shoots, which suggested an increase in the umami taste of tea. On the contrary, flavonoid biosynthesis, especially catechin biosynthesis, was reduced in pea intercropped tea shoots, which prompted the decrease in the bitter taste of tea. In addition, carbohydrate transport and metabolism, and lipid biosynthesis, were obviously different between mono- and intercropped tea plants. These metabolic pathways were closely related to tea quality. 

### 4.1. Pea Intercropping Promoted the Accumulation of Amino Acids in Tea Plants

Amino acids were the important components for evaluating tea quality and could improve the umami taste of tea. In this study, most of the amino acids were increased in pea intercropped tea shoots (Figure 7). *SHMT* catalyzed the reversible conversion between serine and glycine [21]. In pea intercropped tea plants, *SHMT* was upregulated, and there was accumulation of serine and glycine. This result suggests that *SHMT* upregulation was caused by the accumulation of substrates. Similarly, upregulation of five aminoacyl-tRNA biosynthesis-related genes was due to the accumulation of L-glutamine, L-glutamate, and L-asparate. 

There are multiple pathways of amino acid biosynthesis. Carbon is an important source for amino acid biosynthesis [22,23]. Some carbohydrate transport and metabolism-related genes have been proven to contribute to amino acid biosynthesis. For example, a deficiency of plastidial *GAPDH* could decrease the serine content in Arabidopsis [24]. In the present study, *GAPDH* was upregulated in pea intercropped tea plants, which played important roles in accumulation of serine. *PPDK* is also involved in carbohydrate transport and metabolism, and it catalyzes the conversion of pyruvate to phosphoenolpyruvate in plants. Phosphoenolpyruvate is the substrate for the aromatic amino acids, such as phenylalanine, tyrosine, and tryptophan [25]. Thus, *PPDK* upregulation in pea intercropped tea plants provided more substrates for aromatic amino acid synthesis. The G6PGDH catalyzes the last step of the pentose phosphate pathway oxidative phase, generating ribulose-5P and NADPH [26]. While NADPH is the substrate for glutathione synthesis [27,28]. We can therefore suggest that glutathione synthesis is accelerated in pea intercropped tea plants by the expression of G6PGDH, which promotes the accumulation of the substrate NADPH. For glutathione metabolism, *GST* and *GDH* were downregulated in pea intercropped tea plants. Both genes contribute to glutamate synthesis based on substrates of glutathione and NH_3_, respectively [29]. Therefore, glutathione accumulation might also be due to the decreased expression of *GST* and *GDH*. In addition, another glutathione metabolism related gene, *PepN,* was upregulated in pea intercropped tea plants, which catalyzed the glycine synthesis. 

Sulfur is an important macroelement for plant growth and development and is also the source for the biosynthesis of several amino acids, such as cysteine and methionine. There are two sulfur assimilation pathways in plants: one for SO_4_^2−^ reduction and biosynthesis of several amino acids, such as cysteine, methionine, and glutathione; the other one for biosynthesis of adenosine 5′-phosphosulfate (APS) and 3′-phosphoadenosine 5′-phosphosulfate (PAPS), which are the sulfate donors for the sulfation of peptides, hormones, and specialized metabolites, such as phytosulfokine [30]. APS and PAPS were catalyzed by *ASL* and an *ASR*, respectively. In this study, the expression of three *ASRs* and *ASL* was decreased in pea intercropped tea plants compared to monocropped tea plants. In this case, the competition between two sulfur assimilation pathways was reduced. However, no upregulated genes were found in sulfur metabolism. This may be caused by the position of sulfur assimilation, which is located in the root. In general, the inorganic sulfur was absorbed and assimilated into organic compounds in the root, then transported to other plant organs. Similarly, nitrogen absorption and assimilation were conducted in the root, so no nitrogen metabolism related DEGs were identified from pea intercropped tea shoots.

### 4.2. Pea Intercropping Slowed down the Catechin Biosynthesis in Tea Plants

Flavonoids are the major secondary metabolites in tea shoots with strong antioxidant activities [31]. Flavanols, especially catechins, are the richest flavonoids in tea leaves, followed by anthocyanidin and flavonols. Thus, the strong antioxidant activity of tea was endowed by the richest flavonoids. However, catechins contribute to the bitter taste of tea. In pea intercropped tea shoots, the catechins were reduced, which alleviated the bitter taste of tea. 

Aside from contributing to the taste of tea, polyphenols and catechins have antioxidant effects. Catechins were even the target of medicine in the treatment of many diseases, such as coronary heart disease [32], dry eye disease [33], neurodegenerative diseases [34], etc. Thus, the reduction of total polyphenols and catechins might suggest a decreased antioxidant capacity of tea, theoretically. However, different flavonoids possess different degrees of antioxidant activity. For example, to cope with asbestos-induced damage, the protective effects of the flavonoids were: rutin < dihydroquercetin < quercetin < ECG < EGCG [35]. In the present study, EGCG, the richest catechin in tea shoots, did not decrease obviously in pea intercropped tea shoots. In addition, except for catechins, no significant changes were found in other flavonoids, including flavonols and flavones. Therefore, we cannot conclude that a decrease in polyphenols or catechins would reduce the antioxidant activity of tea. In this case, more comparison experiments should be conducted in the future to evaluate the antioxidant activity of different flavonoids. It is not only helpful for people to understand the function of flavonoids but also aids in the development of new therapeutic methods to treat diseases. 

Polyphenols or catechins were synthesized through the flavonoid biosynthesis pathway [36]. In this study, the expression of *ANS*, which is required for C, EC, and EGC biosynthesis, was decreased in pea intercropped tea plants. This may be the cause of the decrease in catechin content. Flavonol synthesis is competitive with catechin biosynthesis, but the expression of *FLS* was also decreased in pea intercropped tea plants. This might be due to the upregulation of *SHCT*, which acts upstream of flavonoid biosynthesis. As a result of the increased *SHCT* expression, the caffeoyl shikimic acid accumulated. However, the substrate for catechin biosynthesis was reduced (Figure 8). Caffeoyl shikimic acid is not only the substrate for caffeoyl CoA and flavonoids but also for caffeic acids and lignins. Therefore, the catechin biosynthesis of pea intercropped tea plants was reduced on account of substrate competition.

Except for amino acid and flavonoid biosynthesis, it is worth noting that many lipids, especially free fatty acids, were increased in pea intercropped tea shoots. Lipids were precursors for many aroma compounds, such as (Z)-3-hexanol (leafy), (E)-2-hexanol, and (E)-2-hexanal (leafy), which gave tea infusions a fresh and greenish aroma [37]. Therefore, pea intercropping might increase the volatile aroma of tea by regulating lipid biosynthesis.

## 5. Conclusions

In conclusion, pea intercropping could promote the umami taste of tea by accelerating the biosynthesis of amino acids by regulating a series of amino acid metabolism-related genes. Additionally, pea intercropping could reduce the bitter taste of tea by slowing down the biosynthesis of flavonoids, especially catechins, through regulating substrate competition upstream of flavonoid biosynthesis.

## Figures and Tables

**Table 1:** Quality of transcriptomic data.

Sample	Total Reads	Mapped Reads	Unique Mapped Reads	GC Content	% ≥ Q30
Tea1	47,836,620	42,016,678 (87.83%)	35,436,061 (74.08%)	45.38%	95.49%
Tea2	53,223,798	46,833,314 (87.99%)	39,326,279 (73.89%)	46.04%	95.21%
Tea3	55,349,774	48,631,380 (87.86%)	40,979,079 (74.04%)	45.80%	95.18%
Pea-tea1	51,540,604	45,241,568 (87.78%)	38,288,792 (74.29%)	45.69%	94.72%
Pea-tea2	58,111,972	50,998,587 (87.76%)	42,914,459 (73.85%)	45.77%	94.89%
Pea-tea3	55,017,918	48,264,631 (87.73%)	40,734,571 (74.04%)	45.35%	95.23%

## Data Availability

The transcriptome data has been deposited in the NCBI SRA database with the accession number PRJNA861687.

## Supplementary Materials

The following supporting information can be downloaded at: https://www.mdpi.com/article/10.3390/foods11223746/s1, Table S1: Primers for quantitative real-time PCR analysis; Table S2: Content of amino acids and derivates in mono- and pea intercropped tea shoots; Table S3 Content of amino acids and derivates in mono- and pea-intercropped tea shoots.
