# Supplementary material for: Pea-Tea Intercropping Improves Tea Quality through Regulating Amino Acid Metabolism and Flavonoid Biosynthesis
Source: Foods. 2022 Nov 21;11(22):3746. doi: 10.3390/foods11223746 (PMC9689014; doi:10.3390/foods11223746)
Supplement: Supplementary file 1 [file foods-11-03746-s001.zip › Table S1.pdf]

Table S1 Primers for quantitative real-time PCR analysis.

Gene ID	Sense primer (F)	Anti-sense primer (R)	Product length (bp)
CSS0028188	ATCTCTATCCTCACCAACTCTTG	TCTTATTCTCCATCGTCTTCTCC	124
CSS0010162	TCTTGGAACCTCACTACTCATAC	ATCTCTAATGTCTGCTTCAACTTG	180
CSS0024005	GCTCGGCGATGATTCCTAAG	AACCAAGTAGATTCCAGTCCTC	139
CSS0032332	GCTGGAAGTGGATATGACAATG	TTTCTGGTCTCCGAGGTAGG	120
CSS0036536	TCTCACCCTTCCCTAACAATGG	TCCTTGCCGATGAACGAGTC	170
CSS0048869	AAACAAGTGAAACCCTGATGG	CTGAGTGAGAGTGAGAGTCC	160
CSS0029656	TCTCAAGCCCATTCCTACAC	GATTGTCAGCAGATAGAGTCG	162
CSS0005382	GTTCCATTACAGACAGCCTCAG	GCTCAACCACCATCTTCACC	151
CsGAPDH	TGGGTGTCAATGAGAAGGATTAC	TTTGTGTGGCTGTGATGGAG	114
